# Antioxidant, carbonic anhydrase inhibition and diuretic activity of *Leptadenia pyrotechnica* Forssk. Decne

**DOI:** 10.1016/j.heliyon.2023.e22485

**Published:** 2023-11-17

**Authors:** Noreena Masood, QurratUlAin Jamil, Muhammad Irfan Aslam, Muhammad Irfan Masood, Jafir Hussain Shirazi, Qazi Adnan Jamil, Muhammad Saeed Jan, Bader Alsuwayt, Ashfaq Ahmad, Sulaiman Mohammed Abdullah Alnasser, Mohammed Aufy, Shahid Muhammad Iqbal

**Affiliations:** a Department of Pharmacology, Faculty of Pharmacy, The Islamia University of Bahawalpur, Bahawalpur 63100, Pakistan; b Department of Pharmacy Practice, Faculty of Pharmacy, The Islamia University of Bahawalpur, Bahawalpur 63100, Pakistan; c Institute of Pharmaceutical Sciences, University of Veterinary and Animal Sciences, Lahore 54000, Pakistan; d Department of Pharmaceutics, Faculty of Pharmacy, The Islamia University of Bahawalpur, Bahawalpur 63100, Pakistan; e Department of Pharmacy, Bacha Khan University, Charsadda 24420, Pakistan; f Department of Pharmacy Practice, College of Pharmacy, University of Hafr Al batin, Hafr Al batin 39524, Saudi Arabia; g Department of Pharmacology and Toxicology, Unaizah College of Pharmacy, Qassim University, Qassim 51452, Saudi Arabia; h Division of Pharmacology and Toxicology, University of Vienna, UZA II, Josef-Holaubek-Platz 2, A-1090, Vienna, Austria

**Keywords:** *Leptadenia pyrotechnica*, GC-MS, Antioxidant, Diuretic, Saluretic, Carbonic anhydrase

## Abstract

**Background:**

*Leptadenia pyrotechnica* Forssk. Decne is a member of family Apocynaceae and locally known as ‘Khipp’. It is found in dry, sandy habitat of Pakistan and in several other regions around the world including Asia, Tropical Africa, Western Gulf and Mediterranean countries. It has nutritional value, containing 4 % lipids, 23 % proteins, 28 % carbohydrates, 4 % fibers, vitamin E and several minerals. Traditionally, this plant has been used by several communities for pain, different inflammatory and kidney disorders. Ethno-botanical studies have reported the use of *L. pyrotechnica* in nephrolithiasis*,* kidney disorders and induction of diuresis, which requires a detailed pharmacological study to validate the folkloric use of *L. pyrotechnica* as diuretic.

**Methods:**

The 70 % methanolic *L. pyrotechnica* (Lp.Cr) extract was prepared and qualitatively checked for the presence of various phytochemicals. Phenolic, flavonoid, tannin and saponin contents were quantified. GC-MS analysis of Lp.Cr was also performed. Antioxidant potential of Lp.Cr was evaluated by DPPH, ABTS and nitrite radical scavenging assays. CUPRAC and FRAP assay described the reducing potential of Lp.Cr. Diuretic activity was performed in both acute and prolonged models at different doses followed by the estimation of electrolytes, urea and creatinine levels. The mechanism of diuresis was described by pre-treatment with atropine, l-NAME, indomethacin and carbonic anhydrase inhibition.

**Results:**

Lp.Cr. indicated high phenolic and flavonoid contents which correlated with good antioxidant activity. GC-MS analysis showed the presence of 104 compounds from different phytochemical classes. Diuretic activity was performed at 10–300 mg/kg concentrations where the dose of 100 and 300 mg/kg showed good diuretic and saluretic activity comparable to furosemide. Lp.Cr exhibited diuresis both in acute and prolonged study protocols which can be attributed to carbonic anhydrase inhibition, effect on prostaglandins and cholinergic pathways.

**Conclusion:**

*L. pyrotechnica* contained several phytochemicals and exhibited good antioxidant activity. It induced diuresis and saluretic activity which was comparable to furosemide at higher doses. Diuretic activity can be attributed to carbonic anhydrase inhibition, prostaglandin synthesis and cholinergic pathways.

## Highlights

• Aerial parts of *L. pyrotechnica* contained 104 phytochemicals identified by GC-MS.
• *L. pyrotechnica* showed good antioxidant activity correlating with total phenolic and flavonoid contents.
• *L. pyrotechnica* induces diuresis and saluresis.
• *L. pyrotechnica* inhibits carbonic anhydrase enzyme.

## Graphical abstract

**Figure:**
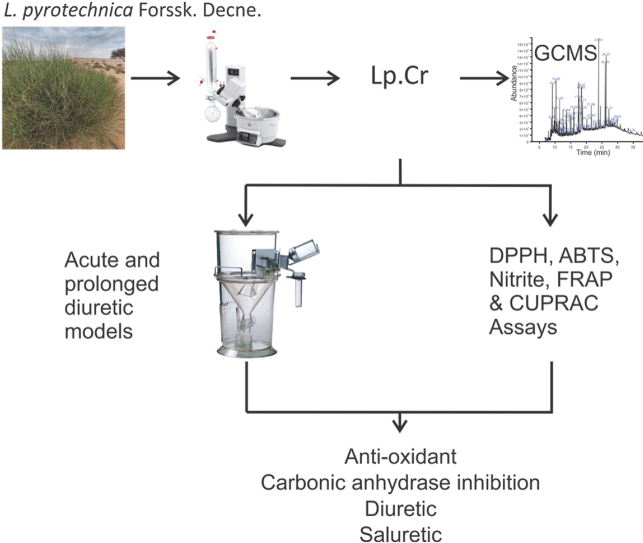

## 1 Introduction

*Leptadenia pyrotechnica* Forssk. Decne. Is a member of family Apocynaceae and widely found in Asia, Africa, Western Gulf and Mediterranean countries. In Pakistan, it is known as ‘Khipp’ and found in a dry and sandy habitat of Cholistan desert of Punjab [1], along the sea coast in Sindh, Baluchistan and in Southern districts of Khyberpakhtunkhwa [2]. It is a nutritious plant and used as a fodder for animals, while its leaves and flower buds are consumed as a vegetable by local community. It contains approximately 4 % lipids, 23 % proteins, 28 % carbohydrates, 4 % fibers, vitamin E, minerals including calcium, phosphorus and iron [3,4]. Several phytochemical classes have been reported in *L. pyrotechnica* including flavonoids, carbohydrates, glycosides, alkaloids, sterols/triterpenes, saponins, coumarins, pyrogallol and catechol tannins [4].

*L. pyrotechnica* is quite rich in phytochemicals and approximately 273 compounds have been identified from various parts of the plant belonging to different chemical classes [4]. It contained 34 terpenes and their derivatives including artemisinin, taraxerol, fernenol, pyrothechnoic acid, squalene, leptadenol, oleanolic acid and seven sterols including β-sitosterol, stigmasterol, campasterol, cholesterol, cucurbitacin E [[4], [5], [6], [7], [8], [9]]. Beside terpenes and sterols, several fatty acids and aromatic hydrocarbons have also been identified [8]. Among glycosides it contained both cardiac and pregnane glycosides [10]. The GC-MS analysis of alkaloid rich extract of *L. pyrotechnica* indicated the presence of pyridine alkaloids while other alkaloids were also present in minor quantities [11]. Among phenolics and flavonoids it contained caffeic acid, gallic acid, vanillic acid, epicatechin, kaempferol-, quercetin-, texacin-flavonoids and cardenolides [[12], [13], [14]]. Phenolic contents of aerial parts of *L. pyrotechnica* has been quantified by HPLC and consisted of caffeic acid, vanillin, vanillic acid, ferulic acid, cinnamic acid, *p*-coumaric acid, veratric acid, myristicin, resorcinol and coumarin in a decreasing order of abundance [15]. It is considered safe for oral consumption as we have already performed and reported acute toxicity studies of 70 % methanolic extract of *L. pyrotechnica* which showed no sign of toxicity after oral administration of 10 g/kg in mice [16], while the sub-chronic toxicity studies performed in Wistar rats indicated the safety of 400 mg/kg oral dose administered for 35 days [17].

Traditionally every part of the plant has a medicinal importance. It is used as an expectorant and have anti-histaminic properties [7]. It is consumed for the management of inflammatory disorders, fever, cough, pain, tumor, asthma, rheumatism, dysmenorrhea, laxative, anabolic, and wound healing [[18], [19], [20], [21]]. Its bark and leaves are used for the preparation of antibacterial, antispasmodic and anti-inflammatory remedies. Aerial parts are consumed by various communities for kidney disorders, kidney pain, and as a diuretic [3,18,22]. Arab Bedouins use infusion of branches for urinary retention and bladder stones [8]. *L. pyrotechnica* has been consumed by several communities as a diuretic and for kidney disorders which requires a detailed pharmacological study to evaluate its diuretic potential [4]. So in the present study we evaluated diuretic activity of 70 % methanolic extract of *L. pyrotechnica* both in acute and prolonged models. Beside diuretic activity we also assessed the underlying diuretic mechanism, carbonic anhydrase inhibition and anti-oxidant activity.

## 2 Materials and methods

### 2.1 Plant collection and preparation of extract

*L. pyrotechnica* whole plant was collected in October 2020, from the Cholistan desert, Punjab, Pakistan (29 38′79.08″ N, 71 76′93.56″). The collected plant was authenticated by a botanist and a sample was submitted to the herbarium of research lab in the Department of Pharmacology, Faculty of Pharmacy with a voucher number LP-WP-08-21-197 for future reference. Plant was cleaned, rinsed and dried in the shade then coarsely powdered. Extraction of L. *pyrotechnica* (Lp.Cr) was performed by 70 % methanol as described previously [23].

### 2.2 Qualitative phytochemical analysis

The presence of alkaloids, coumarins, flavonoids, phenols, tannins, phlobatannins, terpenes, saponins, quinones, resins, glycosides, proteins and amino acids in Lp.Cr were assessed by qualitative methods described previously [24].

### 2.3 Quantitative phytochemical analysis

#### 2.3.1 Total phenolic contents

Total phenolic contents (TPC) of the Lp.Cr were determined by using Folin-Ciocalteau assay [25]. Calibration curve was plotted with gallic acid (7.8–1000 μg/ml). Reaction mixture consisted of 1 ml diluted Folin-Ciocalteau's reagent, 1 ml of Lp.Cr (1 mg/ml), 10 ml sodium carbonate (7 %) and 13 ml double distilled water. Mixture was incubated in the dark for 60 min and absorbance was measured at 750 nm. Total phenolic contents were described as mg gallic acid equivalents per gram of Lp.Cr (mg GAE/g).

#### 2.3.2 Total tannin contents

Tannin contents of Lp.Cr were determined by a method described previously with minor modifications [26]. Total phenolic contents were determined by using tannic acid calibration curve (0.1–1 mg/ml). To precipitate the tannins, 1 ml of Lp.Cr was mixed with 100 mg of polyvinyl polypyrrolidine (PVPP) and 1 ml of distilled water followed by incubation at 4 °C for 4 h. After 4 h it was vortexed and centrifuged at 3000 rpm for 10 min. Supernantant was collected and its phenolic contents were determined again by using Folin-Ciocalteau assay. Tannin contents of the Lp.Cr were calculated by the given formula and expressed as mg tannic acid equivalent per gram of Lp.Cr (mg TAE/g).

Tannins=Totalphenolics−Nonphenolics

#### 2.3.3 Total flavonoid contents

Total flavonoid contents (TFC) of Lp.Cr were determined as described previously [25]. Quercetin (7.8–500 μg/ml) was used to draw the calibration curve. Reaction mixture contained 0.15 ml sodium nitrite (0.5 M) and 0.15 ml aluminum chloride hexahydrate (0.3 M), 3.4 ml 50 % methanol mixed with 0.3 ml Lp.Cr (0.3 mg/ml). After 5 min sodium hydroxide (1 M) was added and then absorbance was measured at 506 nm. Total flavonoid contents were presented as mg quercetin equivalent per gram of Lp.Cr (mg QE/g).

#### 2.3.4 Total saponin contents

Total saponin contents (TSC) of Lp.Cr were determined as described previously (Oluyori et al., 2022). Briefly 1 g of Lp.Cr was mixed with 20 % acetic acid in ethanol and incubated in water bath at 50 °C for 24 h. Reaction mixture was concentrated on water bath followed by dropwise addition of concentrated NH_4_OH until precipitation. Precipitates were allowed to settle down which were filtered and weighed. Saponins were calculated by the formula:

$$\%\text{Saponins}=\frac{\text{weight}\;\text{of}\;\text{dried}\;\text{precipitates}}{\text{weight}\;\text{of}\;\text{sample}}\times 100$$

### 2.4 GC-MS analysis

Lp.Cr was analyzed on GC-MS system (Agilent Technologies, Santa Clara, CA, USA) by preparing the sample as described previously [27]. The 2 μl of sample was injected by maintaining flow rate of helium at 0.8 ml/min. Initially the temperature was kept at 80 °C which was raised to 280 at a rate of 10 °C/min, while the injector temperature was kept at 220 °C. Scanning range was 70–700 *m*/*z* and compounds were identified by comparing relative retention time and mass fragmentation using NIST 2014 mass spectral library [28].

### 2.5 Antioxidant activity

#### 2.5.1 DPPH assay

DPPH radical scavenging assay of Lp.Cr was performed by the procedure described previously [29]. For assay, 150 μl of DPPH (200 mM) was mixed with 50 μl Lp.Cr (1 mg/ml) in a 96 well plate. Different concentrations of trolox (5–60 μg/ml) were used to draw calibration line while 50 μl of methanol was used as a blank. Mixture was incubated for 30 min in dark at room temperature and then absorbance was measured at 517 nm. Experiment was performed in triplicate and described as mg of trolox equivalents per gram of Lp.Cr.

#### 2.5.2 ABTS assay

Total antioxidant capacity of Lp.Cr was determined by ABTS radical cation based assay as described previously with minor modifications [30]. ABTS (7 mM) solution was prepared in water and incubated with potassium persulfate (2.45 mM) in dark for 16 h at room temperature to produce (ABTS**·**^+^) cation. To perform assay the ABTS**·**^+^ cation solution was diluted with ethanol until absorbance of 0.700 ± 0.02 at 734 nm was obtained. 10 μl of Lp.Cr (1 mg/ml) or trolox (5–60 μg/ml) was mixed with 1 ml of ABTS**·**^+^ cation solution and then incubated for 30 min followed by the measurement of absorbance at 734 nm. Experiment was performed in triplicate and results were described as mg of trolox equivalents per gram of Lp.Cr.

#### 2.5.3 Nitrite scavenging assay

Nitrite scavenging activity of Lp.Cr was performed as described previously [31]. Lp.Cr or ascorbic acid (0.125–2.0 mg/ml) were mixed with 1 ml of NaNO_2_ (1 mM) and pH 2.0 was adjusted with 0.1 N HCl. Volume of reaction mixture was adjusted to 10 ml with distilled water and incubated for 1 h at 37 °C. After incubation, reaction mixture 100 μl was mixed with 500 μl of distilled water and then 100 μl of Griess reagent was added. It was again incubated for 15 min at room temperature and absorbance was measured at 540 nm. Scavenging activity was calculated by the formula:

$$\%\text{Scavenging}=100-\left[\left\{\frac{\left(\text{Abs}\;\text{with}\;\text{Griess}\;\text{reagent}-\text{Abs}\;\text{without}\;\text{Griess}\;\text{reagent}\right)}{\text{Abs}\;\text{of}\;\text{control}}\right\}\times 100\right]$$

#### 2.5.4 CUPRAC assay

Lp.Cr was assessed for total antioxidant activity by cupric ion reducing antioxidant capacity (CUPRAC) assay as described previously with minor modifications [32]. CUPRAC reagent was prepared by mixing 1 ml CuCl_2_.2H_2_O (10 mM), 1 ml neocuproine (7.5 mM in 96 % ethanol) and 1 ml ammonium acetate buffer (pH 7). For CUPRAC assay 1 ml of Lp.Cr (1 mg/ml) or trolox (5–60 μg/ml) was mixed with 3 ml of CUPRAC reagent and incubated for 1 h at room temperature then absorbance was measured at 450 nm. Experiment was performed in triplicate and reductive capacity of Lp.Cr was reported as mg of trolox equivalents per gram of Lp.Cr.

#### 2.5.5 FRAP assay

Ferric ion reducing antioxidant power (FRAP) of Lp.Cr was determied by previously described method with minor modifications [33]. FRAP reagent was prepared with 25 ml acetate buffer (300 mmol/L; pH 3.6), 2.5 ml TPTZ (10 mmol/L) in HCl (40 mmol/L) and 2.5 ml FeCl_3_.6H_2_O (20 mmol/L). 10 μl of Lp.Cr (1 mg/ml) or trolox (5–60 μg/ml) was vortexed with 30 μl water and 300 μl of freshly prepared FRAP reagent. A blank was prepared similarly with water. Mixture of Lp.Cr, trolox and blank were incubated at room temperature for 30 min then absorbance was measured at 593 nm. Experiment was performed in triplicate and results were described as mg of trolox equivalents per gram of Lp.Cr.

### 2.6 Diuretic assay

#### 2.6.1 Animals

Male Wistar Albino rats weighing 200–250 g were kept in the animal house, of Department of Pharmacology, Faculty of Pharmacy, the Islamia University of Bahawalpur, Pakistan. Animals were housed in wooden cages, six animals per cage at standard temperature and humidity. Rats were given pelleted diet and free access to water while maintaining 12 h light-dark cycle. Institutional animal ethics committee approved the study protocol vide reference no. PAEC/21/5.

#### 2.6.2 Acute diuretic activity and involvement of prostaglandins, cholinergic and NO-pathway

Acute diuretic activity of Lp.Cr was performed on male Wistar rats by following the method described previously with minor modifications [34]. Animals were grouped with six animals in each group. Group-I (control group), received 10 ml/kg normal saline only, Group-II received furosemide 10 mg/kg while Group-III, IV, V and VI were treatment groups received Lp.Cr 10, 30, 100 and 300 mg/kg p.o. respectively. Animals were fasted overnight before experiment with free access to water and acclimatized for 2 h in the metabolic cages. Bladder of animals was emptied by pulling the tail and gently compressing the pelvic area. Treatments were administered to the animals according to designated groups and placed into the metabolic cages. In additional experiments to assess the underlying mechanism of action, animals were pre-treated for 1 h with 60 mg/kg N-nitroarginine methyl ester (l-NAME), or 1 mg/kg atropine or 5 mg/kg indomethacin [35], followed by the administration of Lp.Cr 100 mg/kg and were placed in the metabolic cages. Volume of urine was measured at 1st hour and then every 2 h up to the 7th hour and then finally at 24th hour. Urine was collected, filtered and cumulative volume was measured. The pH of urine sample was measured and urine samples were stored at −20 °C for further analysis. Urine output of all groups was calculated in relation to the body weight of rats and expressed as ml/100 g of animal weight.

#### 2.6.3 Prolonged diuretic activity

Prolonged diuretic activity of Lp.Cr was assessed by previously described method with minor modifications [36]. Animals were divided into three groups (n = 6). Group-I (control group), received 10 ml/kg normal saline only, Group-II received furosemide 10 mg/kg while Group-III received Lp.Cr 100 mg/kg for continuous seven days. Urine volume was measured with graduated cylinder on 1st and 7th day.

#### 2.6.4 Measurement of urinary parameters

Different urinary parameters including diuretic index, Lipschitz value, saluretic and naturetic index was calculated described previously [37] by using following formulas:

$$\text{Diuretic}\;\text{index}=\frac{\text{mean}\;\text{urine}\;\text{volume}\;\text{of}\;\text{test}\;\text{group}}{\text{mean}\;\text{urine}\;\text{volume}\;\text{of}\;\text{control}\;\text{group}}$$

$$\text{Lipschitz}\;\text{value}=\frac{\text{mean}\;\text{urine}\;\text{volume}\;\text{of}\;\text{test}\;\text{group}}{\text{mean}\;\text{urine}\;\text{volume}\;\text{of}\;\text{Standarad}\;\text{group}}$$

$$\text{Saluretic}\;\text{index}=\frac{\text{electrolyte}\;\text{conc}\;\text{in}\;\text{test}\;\text{group}\;\text{urine}}{\text{electrolyte}\;\text{conc}\;\text{in}\;\text{control}\;\text{group}\;\text{urine}}$$

$$\text{Naturetic}\;\text{index}=\frac{\text{urinary}\;\text{excretion}\;\text{of}\;\text{sodium}}{\text{urinary}\;\text{excretion}\;\text{of}\;\text{potassium}}$$

#### 2.6.5 Determination of electrolyte, urea and creatinine levels

Urinary Na^+^, K^+^ and Cl^−^ levels were measured by SPOTCHEM EL analyzer (arkray global business, Inc. Japan), while urea and creatinine were measured by commercially available kits according to manufacturer protocol (AMS Srl., Italy) on Microlab 300 (ELITechGroup, France).

### 2.7 Carbonic anhydrase inhibitory activity

Carbonic anhydrase inhibition by Lp.Cr was assessed as described previously with minor modifications [38]. Assay mixture was made by mixing 120 μl of 50 mM Tris buffer containing 0.1 mM ZnCl_2_ (pH 7.6), 20 μl of test substance and 20 μl of bovine carbonic anhydrase (50 U). Mixture was shaked and incubated for 10 min at room temperature. After incubation a 40 μl of substrate *p*-nitrophenyl acetate (6 mM stock prepared freshly), was added to achieve final concentration of 0.6 mM per well and incubated for 30 min at room temperature followed by measurement of absorbance at 348 nm. Acetazolamide was used as standard and %-inhibition was calculated by given formula:

$$\%-\text{Inhibition}=\left[\frac{\left(\text{Blank}-\text{Sample}\right)}{\text{Blank}}\right]\times 100$$

### 2.8 Statistical analysis

Results were analyzed for statistical significance by GraphPad Prism version 8 (GraphPad Software, San Diego, CA, USA), and described as Mean ± SEM. Significance was calculated by one-way and two-way ANOVA followed by Tukey's or Dunnett's multiple comparison test where appropriate; p value < 0.05 was considered as statistically significant.

## 3 Results

### 3.1 Plant collection and extraction

Approximately 800 g of *L. pyrotechnica* was extracted with 70 % aqueous methanol. Weight of dried *L. pyrotechnica* crude extract (Lp.Cr) was 65.8 g, having an approximate yield of 8.22 %.

### 3.2 Qualitative phytochemical analysis

Preliminary phytochemical analysis of Lp.Cr indicated the presence of various phytochemicals described in Table 1.

**Table 1:** Qualitative analysis of Lp.Cr for the presence ‘+’ or absence ‘-’ of various phytochemicals.

Phytochemical	Detection
Alkaloids	+
Phenols	+
Tannins	+
Flavonoids	+
Saponins	+
Glycosides	+
Terpenes	+
Coumarins	+
Proteins and aminoacids	+
Quinones	–
Resins	–
Phlobatannins	–

### 3.3 Quantitative phytochemical analysis

#### 3.3.1 Total phenolic contents

Total phenolic contents of Lp.Cr were assessed by Folin-Ciocalteu method where gallic acid was used as a standard and estimated as 54.52 ± 9.22 mg GAE/g dry weight of the Lp.Cr.

#### 3.3.2 Total tannin contents

Total tannin contents of Lp.Cr were assessed by Folin-Ciocalteu reagent by using tannic acid as standard. Phenolic contents before and after precipitation of tannins with PVPP were assessed and estimated as 0.54 ± 0.01 mg TAE/g dry weight of the Lp.Cr.

#### 3.3.3 Total flavonoid contents

Total flavonoid contents in Lp.Cr were estimated as 35 ± 11.78 mg QE/g dry weight of the Lp.Cr.

#### 3.3.4 Total saponin contents

Total saponin contents of Lp.Cr were also estimated, which showed 1 g of Lp.Cr contained 7.93 ± 0.85 % saponins.

### 3.4 GC-MS analysis

**Fig. 1:**
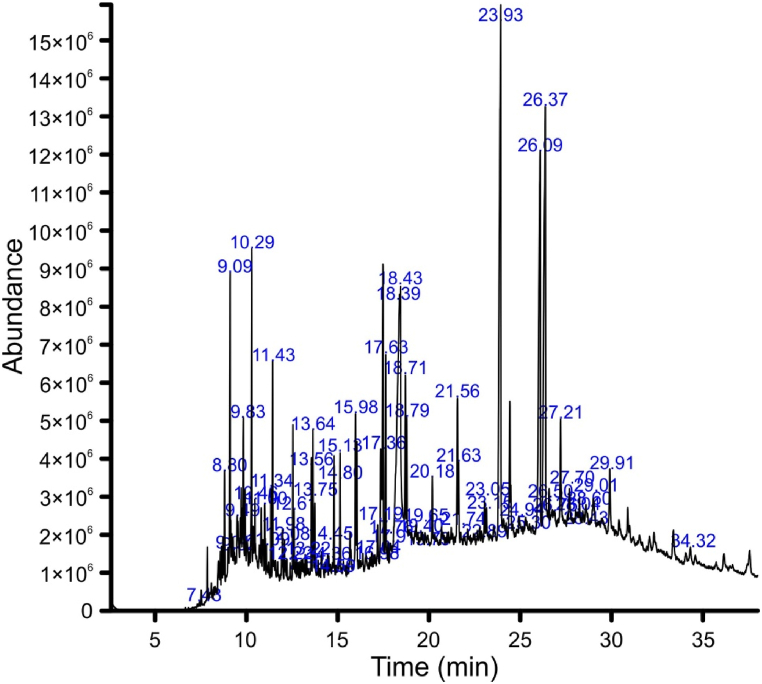
Complete GC-MS chromatogram of Lp.Cr showing the peaks of phytochemicals identified by correlating *m*/*z* with NIST-11 library.

GC-MS analysis of Lp.Cr indicated the presence of 104 compounds from various chemical classes including alkanes, alkenes, organic metalloid salts, sterols, esters, long chain and branched chain fatty acids. Among these, 64 compounds showed quality factor greater than 90 which are presented in Table 2, while the complete GC-MS chromatogram is shown in Fig. 1. The table describing total identified compounds is included in supplementary material.

**Table 2:** GC-MS analysis of Lp.Cr described with peak number, retention time, molecular formula, molecular weight and quality factor.

Sr.No	Peak no	RT	Area%	Compound name	M.F.	M.W. g/mol	Qual
1	1	7.43	0.04	2-methyldodecane	C_13_H_28_	184.3	93
2	12	8.80	0.58	2,6,10-trimethyldodecane	C_15_H_32_	212.4	91
3	13	9.01	0.79	Ethylcyclododecane	C_14_H_28_	196.3	92
4	14	9.09	1.85	Tetradecane	C_14_H_30_	198.3	98
5	17	9.32	0.42	4-methoxy-6-fluoro- benzyl alcohol	C_8_H_9_FO_2_	156.1	90
6	18	9.49	1.57	2,3-dimethylnaphthalene	C_12_H_12_	156.2	96
7	20	9.83	1.39	Tridecane	C_13_H_28_	184.3	90
8	24	10.29	1.85	Pentadecane	C_15_H_32_	212.4	97
9	26	10.46	1.27	2,4-ditert-butylphenol	C_14_H_22_O	206.3	95
10	30	11.00	0.55	1,6,7-trimethylnaphthalene	C_13_H_14_	170.2	96
11	31	11.09	0.36	3-methylpentadecane	C_16_H_34_	226.4	96
12	33	11.34	0.46	Pentadecyl 2-chloroacetate	C_17_H_33_ClO_2_	304.9	94
13	34	11.43	1.17	Hexadecane	C_16_H_34_	226.4	99
14	37	11.98	0.67	2-bromo dodecane	C_12_H_25_Br	249.2	95
15	38	12.08	0.45	2-benzylideneheptanal	C_14_H_18_O	202.2	97
16	39	12.22	0.22	3-methylhexadecane	C_17_H_36_	240.5	94
17	42	12.61	0.47	2,6,10,14-tetramethylpentadecane	C_19_H_40_	268.5	93
18	44	12.82	0.25	2-methyl-Z-4-tetradecene	C_15_H_30_	210.4	90
19	49	13.22	0.2	2-methylheptadecane	C_18_H_38_	254.5	96
20	52	13.56	0.56	1-octadecene	C_18_H_36_	252.5	95
21	53	13.64	0.77	Octadecane	C_18_H_38_	254.5	98
22	54	13.75	0.48	3-methylheptadecane	C_18_H_38_	254.5	90
23	60	14.36	0.17	Cembrane	C_20_H_40_	280.5	94
24	61	14.45	0.17	3-methyloctadecane	C_19_H_40_	268.5	91
25	63	14.59	0.18	1-eicosene	C_20_H_40_	280.5	90
26	65	14.80	0.57	Nonadecane	C_19_H_40_	268.5	97
27	67	15.13	0.78	Methyl hexadecanoate	C_17_H_34_O_2_	270.5	98
28	74	15.98	0.89	5-eicosene	C_20_H_40_	280.5	98
29	83	16.98	0.13	Cycloeicosane	C_20_H_40_	280.5	93
30	84	17.04	0.17	2,6,10,14-tetramethyl-hexadecane	C_20_H_42_	282.5	91
31	85	17.19	0.46	*cis*-1-chloro-9-octadecene	C_18_H_35_Cl	286.9	98
32	86	17.36	1.18	Methyl lineoleate	C_19_H_34_O_2_	294.5	99
33	88	17.63	1.28	Phytol	C_20_H_40_O	296.5	94
34	89	17.76	0.19	Methyl octadecanoate	C_19_H_38_O_2_	298.5	95
35	90	17.90	0.41	1-chloro-octadecane	C_18_H_37_Cl	288.9	91
36	91	18.39	6.44	Methyl alpha-linolenate	C_19_H_32_O_2_	292.5	91
37	92	18.43	2.73	Linolenyl alcohol	C_18_H_32_O	264.4	93
38	93	18.71	1.33	1-docosene	C_22_H_44_	308.6	94
39	94	18.79	0.98	Eicosane	C_20_H_42_	282.5	97
40	97	19.41	0.28	2-methyl-octadecane	C_19_H_40_	268.5	91
41	99	19.65	0.39	Docosane	C_22_H_46_	310.6	95
42	100	19.79	0.42	Heneicosane	C_21_H_44_	296.6	96
43	102	20.18	0.96	Heptadecane	C_17_H_36_	240.5	96
44	112	21.56	1.19	Cyclotetracosane	C_24_H_48_	336.6	99
45	113	21.63	0.6	Tetracosane	C_24_H_50_	338.7	98
46	114	21.74	0.38	Tricosane	C_23_H_48_	324.6	93
47	123	22.89	0.28	2,6,10,14,18-eicosapentaene	C_25_H_42_	342.6	90
48	124	23.05	0.49	Pentacosane	C_25_H_52_	352.7	98
49	125	23.15	0.57	Erucic acid	C_22_H_42_O_2_	338.6	91
50	127	23.93	6.74	2-ethylhexyl hydrogen phthalate	C_16_H_22_O_4_	278.3	91
51	132	24.94	0.97	Hexacosane	C_26_H_54_	366.7	94
52	135	25.30	0.63	1-hexacosene	C_26_H_52_	364.7	95
53	138	26.09	5.13	*Cis*-permethrin	C_21_H_20_Cl_2_O_3_	391.3	99
54	139	26.37	5.77	*Trans*-permethrin	C_21_H_20_Cl_2_O_3_	391.3	99
55	140	26.5	0.23	Cyclohexylbis[5-methyl-2-(1-methylethyl)cyclohexyl]- phosphine	C_26_H_49_P	392.6	91
56	142	26.76	0.51	Cyclotriacontane	C_30_H_60_	420.8	96
57	144	27.21	1.98	1-nonadecene	C_19_H_38_	266.5	98
58	146	27.70	0.71	1,54-dibromotetrapentacontane	C_54_H_108_Br_2_	917.2	90
59	147	28.04	0.4	1-(4-Bromobutyl)-2-piperidinone	C_9_H_16_BrNO	234.1	91
60	150	28.44	0.27	2-dodecen-1-ylsuccinic anhydride	C_16_H_26_O_3_	266.3	93
61	151	28.60	0.68	Heptacosane	C_27_H_56_	380.7	93
62	152	29.01	0.99	Octacosane	C_28_H_58_	394.8	95
63	154	29.91	0.6	Octacosanol	C_28_H_58_O	410.8	95
64	163	34.32	0.26	Pyridine-3-carboxamide	C_13_H_10_F_3_N_3_O	281.2	91

### 3.5 Antioxidant assays

#### 3.5.1 DPPH assay

Free radical scavenging activity of Lp.Cr was assessed by DPPH assay by using trolox for comparison. The DPPH free radical scavenging activity of Lp.Cr was estimated as 28.68 ± 0.34 mg trolox equivalents per gram of Lp.Cr (mg TE/g).

#### 3.5.2 ABTS assay

Free radical scavenging activity of Lp.Cr was also assessed by ABTS radical scavening assay and trolox was used for comparison. ABTS radical scavenging activity of Lp.Cr was estimated as 23.54 ± 1.48 mg TE/g of Lp.Cr.

#### 3.5.3 Nitrite scavenging assay

Nitrite ion scavenging activity of Lp.Cr was assessed by Griess reagent. Ascorbic acid 0.01–2.0 mg/ml was used as a standard which showed 16.75 ± 2.53–79.49 ± 0.29 % scavenging activity with the IC_50_ value of 0.56 ± 0.08 mg/ml. Lp.Cr 31.25–50 mg/ml showed 7.16 ± 0.45–71.1 ± 3.25 % scavenging activity with the IC_50_ value of 17.02 ± 2.12 mg/ml.

#### 3.5.4 CUPRAC assay

Total antioxidant capacity of Lp.Cr was assessed by CUPRAC assay. Calibration curve of trolox (5–60 μg/ml) was constructed for estimation. CUPRAC assay described the total antioxidant capacity of Lp.Cr as 161.22 ± 9.42 mg TE/g of Lp.Cr.

#### 3.5.5 FRAP assay

Reducing potential of Lp.Cr was assessed by the FRAP assay, which measures the ability to reduce Fe^3+^-tripyridltriazine to Fe^2+^-tripyridltriazine. Calibration curve of trolox (5–60 μg/ml) was constructed for estimation. FRAP assay described the reducing potential of Lp.Cr as 31.59 ± 1.63 mg TE/g of Lp.Cr.

### 3.6 Diuretic assay

#### 3.6.1 Acute diuretic activity and involvement of prostaglandins, cholinergic and NO-pathway

**Fig. 2:**
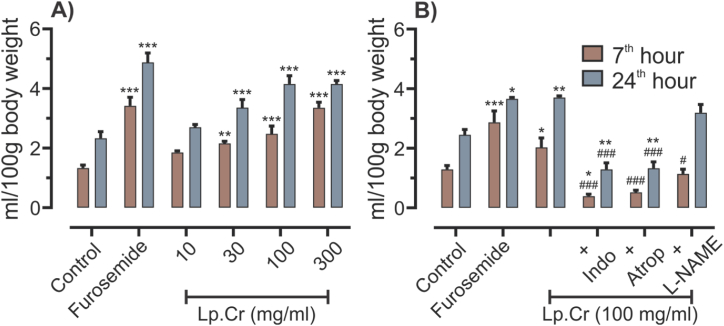
Effect of Lp.Cr on diuresis. Values are expressed as Mean ± SEM, n = 6 per group. Results are analyzed by using two way ANOVA followed by Dunnett's multiple comparison test. A) Lp.Cr treatments and furosemide were compared with the normal control group and significance was denoted by (*). B) Treatment groups were compared with the control group and significance was denoted by (*), while treatment groups co-administered with indomethacin, atropine and l-NAME were also compared with Lp.Cr 100 mg/kg treatment and significance was denoted (#). The results were considered statistically significant (*/#) if P < 0.05, (**/##) if P < 0.01 and (***/###) if P < 0.001.

Diuretic activity of Lp.Cr was assessed in male Wistar rats for four different concentrations i.e. 10, 30, 100, 300 mg/kg and was compared to the control group (normal saline) and standard group (furosemide 10 mg/kg). Volume of urine was measured at 1st, 3rd, 5th, 7th and 24th hour and expressed in relation to body weight as ml/100 g. Diuretic activity of Lp.Cr was gradually developed. On comparison with normal control at 1st hour no statistically significant difference was observed between Lp.Cr treatment groups. At 3rd hour, only Lp.Cr 300 mg/kg treatment group produced significantly increased diuresis compared to control group with a value of 1.08 ± 0.08 ml (p < 0.05). At the 5th hour Lp.Cr treatment groups 30, 100 and 300 mg/kg showed significantly increased urine output compared to the control group with values of 1.49 ± 0.03 (p < 0.05), 1.49 ± 0.10 (p < 0.05) and 2.51 ± 0.14 ml (p < 0.001) respectively. This trend of increase in urine output continued in 7th hour also where the urine output was 2.16 ± 0.07 (p < 0.01), 2.48 ± 0.26 (p < 0.01) and 3.35 ± 0.19 ml (p < 0.001) for Lp.Cr treatment groups 30, 100 and 300 mg/kg respectively, when compared to the control group. Diuretic activity of Lp.Cr maintained until 24th hour where the increase in urine output of Lp.Cr 30, 100 and 300 mg/kg were statistically significant (p < 0.001), compared to the control group. When the Lp.Cr treatment groups were compared to the furosemide, only the Lp.Cr 300 mg/kg treatment group showed insignificant difference at 5th, 7th and 24th hour indicating that the diuretic activity of Lp.Cr 300 mg/kg treatment was comparable to the furosemide 10 mg/kg (Table 3; Fig. 2A). The diuretic index and Lipschitz value at 7th and 24th hour were calculated for all treatment groups and are presented in Table 5.

**Table 3:** Effect of Lp.Cr on diuresis.

Time (h)	Urine volume (ml)/100g body weight					
	Control	Standard	Treatment			
	NS (10 ml/kg)	Furosemide (10 mg/kg)	Lp.Cr (mg/kg)			
			10	30	100	300
1	0.15 ± 0.07	0.85 ± 0.07**	0.08 ± 0.02	0.24 ± 0.07	0.39 ± 0.06	0.53 ± 0.09
3	0.50 ± 0.09	1.72 ± 0.19***	0.68 ± 0.07	0.93 ± 0.06	0.93 ± 0.06	1.08 ± 0.08*
5	0.83 ± 0.11	2.62 ± 0.24***	1.04 ± 0.11	1.49 ± 0.03*	1.49 ± 0.10*	2.51 ± 0.14***
7	1.33 ± 0.10	3.42 ± 0.29***	1.85 ± 0.06	2.16 ± 0.07**	2.48 ± 0.26***	3.35 ± 0.19***
24	2.33 ± 0.22	4.88 ± 0.31***	2.70 ± 0.09	3.36 ± 0.27***	4.15 ± 0.28***	4.15 ± 0.12***

Results were analyzed by using two way ANOVA followed by Dunnett's multiple comparison test. All treatment groups were compared with control group and significance was denoted (*) if P < 0.05, (**) if P < 0.01 and (***) if P < 0.001.

To assess the involvement of cholinergic, prostaglandins and NO-pathway, animals were pre-treated for 1 h with 1 mg/kg atropine or 5 mg/kg indomethacin or 60 mg/kg L-NAME respectively, followed by the administration of Lp.Cr 100 mg/kg. Co-administration of atropine and indomethacin with Lp.Cr 100 mg/kg significantly reduced the urine output both at 7th and 24th hour, while the co-administration of l-NAME reduced the urine output at 7th hour but no significant difference was observed at 24th hour compared to the Lp.Cr 100 mg/kg when administered alone (Fig. 2B).

#### 3.6.2 Prolonged diuretic activity

**Fig. 3:**
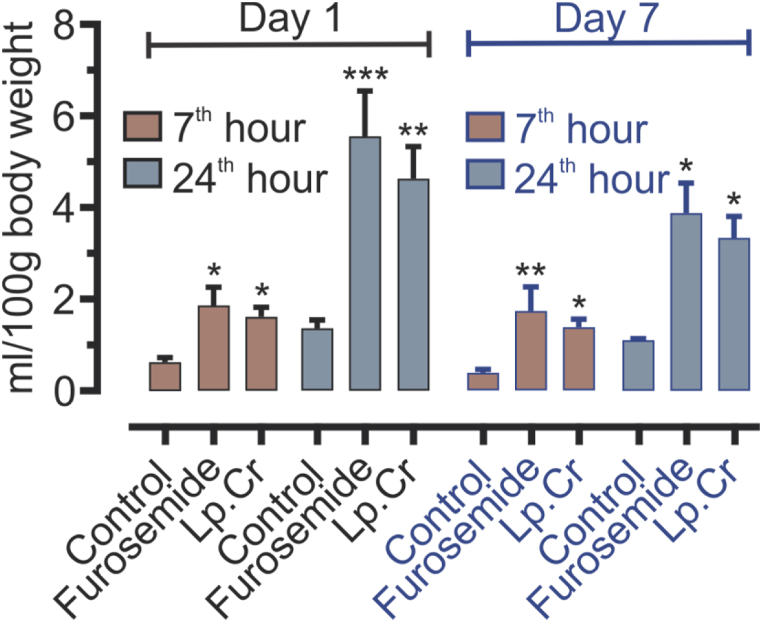
Diuretic effect of Lp.Cr 100 mg/kg through a 7-day continuous treatment model. Values are expressed as Mean ± SEM, n = 6 per group. Results are analyzed by using two way ANOVA followed by Dunnett's multiple comparison test. Lp.Cr 100 mg/kg and furosemide 10 mg/kg treatments were compared with the control group on same day and time point. Statistical significance was denoted by (*), and described as (*) if P < 0.05, (**) if P < 0.01 and (***) if P < 0.001.

Diuretic activity of Lp.Cr was also assessed by 7-day continuous treatment model. Animals were divided into three different groups where control group received normal saline (10 ml/kg), while the other two groups received furosemide (10 mg/kg) and Lp.Cr (100 mg/kg). Urine volume was measured on the 1st and 7th day at two time points i.e. 7th and 24th hour (Fig. 3).

Urine output on day-1 was significantly increased at 7th and 24th hour in both furosemide and Lp.Cr (100 mg/kg) treatment groups. Urine output in Lp.Cr 100 mg/kg treatment group was 1.61 ± 0.20 and 4.63 ± 0.69 ml/100g body weight, at 7th and 24th hour respectively. This increase in urine output was statistically significant until 7th day of the treatment. On day-7, urine output of Lp.Cr 100 mg/kg treatment group was 1.38 ± 0.17 and 3.34 ± 0.46 ml/100g body weight, at 7th and 24th hour respectively.

#### 3.6.3 Measurement of urinary Na^+^, K^+^, Cl^−^

Electrolytes concentration in 24 h urine samples of all groups was determined. Urinary sodium excretion in the control group was 127.7 ± 16.8 mmol/l and furosemide group excreted 210.4 ± 11.2 mmol/l. Test groups of Lp.Cr 10, 30, 100 and 300 mg/kg exhibited urinary sodium concentrations of 146.0 ± 11.0, 171.0 ± 4.0, 199.0 ± 13.9 and 217.0 ± 1.0 mmol/l respectively. When compared to the control group, Lp.Cr 100 and 300 mg/kg demonstrated a significant increase in the urinary sodium excretion. Lp.Cr 300 mg/kg treatment group caused enhanced urinary sodium excretion compared to that of furosemide 10 mg/kg (Table 4).

**Table 4:** Effects of Lp.Cr on urinary electrolyte excretion, naturetic and saluretic index.

Treatment (mg/kg)	Na^+^ (mmol/L)	K^+^ (mmol/L)	Cl^−^ (mmol/L)	Na^+^/k^+^	Saluretic index		
					Na^+^	K^+^	Cl^−^
Control (N/S)	127.7 ± 16.8	46.2 ± 3.1	80.8 ± 3.1	2.76	1.00	1.00	1.00
Furosemide	210.4 ± 11.2***	71.9 ± 2.7***	138.4 ± 14.2**	2.66	1.65	1.56	1.71
Lp.Cr 10	146.0 ± 11.0	60.4 ± 1.8*	122.8 ± 3.1	2.42	1.14	1.31	1.52
Lp.Cr 30	171.0 ± 4.0	69.8 ± 1.2***	139.8 ± 1.2**	2.45	1.34	1.51	1.73
Lp.Cr 100	199.0 ± 13.9***	71.0 ± 1.5***	133.6 ± 7.0**	2.80	1.56	1.54	1.65
Lp.Cr 300	217.0 ± 1.0***	79.0 ± 0. 9***	155.0 ± 6.4**	2.75	1.70	1.71	1.92
Lp.Cr 100 + Indomethacin	231.3 ± 2.3***	80.7 ± 5.0***	159.5 ± 18.3***	2.87	1.81	1.75	1.97
Lp.Cr 100 + Atropine	212.5 ± 4.6***	78.2 ± 0.8***	112.8 ± 5.5	2.72	1.66	1.69	1.41
Lp.Cr 100 + l-NAME	224.0 ± 8.9***	74.6 ± 0.6***	137.9 ± 8.6**	3.0	1.75	1.61	1.71

Results were analyzed by using two way ANOVA followed by Dunnett's multiple comparison test. All treatment groups were compared with control group and significance was denoted (*) if P < 0.05, (**) if P < 0.01 and (***) if P < 0.001.

**Table 5:** Effects of Lp.Cr on urinary pH, creatinine, urea, Lipschitz value and diuretic index.

Treatment (mg/kg)	Lipschitz value	Diuretic index	pH	Creatinine mg/dl/24h	Urea mg/dl/24h
Control (N/S)	0.47	1.00	7.77 ± 0.04	25.13 ± 0.37	27.44 ± 1.18
Furosemide	1.00	2.09	8.82 ± 0.04	21.11 ± 7.04	16.68 ± 7.17
Lp.Cr 10	0.55	1.16	7.85 ± 0.02	23.06 ± 1.15	23.04 ± 0.75
Lp.Cr 30	0.69	1.44	7.85 ± 0.07	21.69 ± 2.18	21.62 ± 2.33
Lp.Cr 100	0.85	1.78	8.63 ± 0.04	22.02 ± 7.19	21.53 ± 4.64
Lp.Cr 300	0.85	1.78	8.67 ± 0.08	21.66 ± 5.78	20.68 ± 6.78
Lp.Cr 100 + Indomethacin	0.35	0.53	8.41 ± 0.03	13.75 ± 1.25	14.76 ± 3.04
Lp.Cr 100 + Atropine	0.36	0.54	8.54 ± 0.02	20.00 ± 6.28	16.09 ± 2.93
Lp.Cr 100 + l-NAME	0.87	1.30	8.33 ± 0.07	15.00 ± 2.50	15.16 ± 3.81

Results were analyzed by using two way ANOVA followed by Dunnett's multiple comparison test. Creatinine and urea excretion for all treatment groups were compared with control group and significance was denoted (*) if P < 0.05, (**) if P < 0.01 and (***) if P < 0.001.

Urinary potassium excretion of control group was 46.2 ± 3.1 whereas Lp.Cr 10, 30, 100 and 300 mg/kg excreted potassium 60.4 ± 1.8, 69.8 ± 1.2, 71.0 ± 1.5 and 79.0 ± 0.9 mmol/l respectively. It indicated that Lp.Cr treated groups had considerably greater urinary potassium concentrations than control group. Potassium level in the urine of the furosemide group was 71.9 ± 2.7 mmol/l, which was comparable to Lp.Cr 300 mg/kg treatment group (Table 4).

Urinary chloride ion concentration of control group was 80.8 ± 3.1 mmol/l, was considerably higher in furosemide and Lp.Cr treated groups (Table 3). The rate of increase in chloride excretion was dose dependent as Lp.Cr 10, 30, 100 and 300 mg/kg indicated urinary chloride 122.8 ± 3.1, 139.8 ± 1.2, 133.6 ± 7.0 and 155.0 ± 6.4 mmol/l respectively. Rate of chloride excretion of Lp.Cr 300 mg/kg was comparable to the furosemide treatment group (Table 4).

Natriuretic index of treatment groups was calculated from the ratio of urinary sodium to potassium excretion of the same group. Lp.Cr 10 and 30 mg/kg had lesser natriuretic action than furosemide, but 100 and 300 mg/kg showed equal natriuretic activity.

Saluretic activity of Lp.Cr 10, 30 and 100 mg/kg was 1.14, 1.34 and 1.56 respectively, as measured by saluretic index for sodium, whereas the saluretic index for potassium was 1.31, 1.51 and 1.54. Saluretic index for chloride was 1.52, 1.73, 1.65 and 1.92 for Lp.Cr 10, 30, 100 and 300 mg/kg respectively. When compared to furosemide, Lp.Cr 300 mg/kg showed greater natriuretic and kaliuretic action (Table 4).

#### 3.6.4 Measurement of urinary pH, urea and creatinine

After 24 h total urine collection, pH of urine sample was measured by using pH meter (pH 720; inoLab). Control group demonstrated pH value 7.77 ± 0.04 whereas the furosemide group showed pH of 8.82 ± 0.04, which was more alkaline than control group. Urine pH data presented in Table 4, indicate that both furosemide and Lp.Cr produced more alkaline urine than control group. Urine creatinine and urea were also quantified for all experimental groups which showed statistically insignificant changes among all treatment groups (Table 5).

### 3.7 Carbonic anhydrase inhibitory activity

Lp.Cr showed marked carbonic anhydrase inhibitory activity with IC_50_ value of 2.89 μg/ml. Acetazolamide was used as a standard which exhibited IC_50_ of 1.71 μg/ml.

## 4 Discussion

Diuretics are the substances that increase urinary volume and facilitate electrolyte excretion. They do so by promoting removal of excess water from extracellular fluid and inhibit sodium reabsorption in nephrons. They are used in a variety of conditions like edema, cardiovascular disorders (CHF, hypertension and stroke), ascites effusion, liver cirrhosis and kidney disorders. Diuretics induce and maintain increased natriuresis until euvolaemia is achieved after which dietary intake of sodium and water is reduced to maintain homeostasis. Conventional diuretics such as thiazide and loop diuretics are used to treat these conditions, but their long term use is associated with several adverse effects including electrolyte imbalance, hyperglycemia, acid-base imbalance, arrhythmias, metabolic disturbance and acute hypovolemia [39].

*L. pyrotechnica* has been studied pharmacologically which exhibited hypolipidemic [20], hepatoprotective [40], anti-diabetic [41], antioxidant [42,43], analgesic, anti-inflammatory [44,45] and anti-diarrheal activity [46]. It is immunostimulant in nature and improves neutrophil adhesion and phagocytic index [16]. It inhibits several enzymes including acetylcholinesterase, buterylcholinesterase, tyrosinase, α-amylase and α-glucosidase [47,48]. The hexane extract of *L. pyrotechnica* exhibited anti-cancer effects by inducing apoptosis in colon cancer cells through caspase activation and p53 pathway [49]. It possesses antibacterial activity against *S. aureus*, *S. epidermidis, B. subtilis* and *P. aeruginosa* [50,51]. Its ethanolic extract increased testosterone production and improved the fertility parameters [52]. In addition to these reported pharmacological activities, *L. pyrotechnica* has been used traditionally by various communities for kidney disorders, bladder stones and urinary retention [3,8,18,22]. Despite these reported traditional uses, we did not find any detailed pharmacological study describing the diuretic effect of *L. pyrotechnica* [4]. A recent study preliminary screened the methnolic extract of *L. pyrotechnica* through Lipschitz test which described good diuretic, natriuretic and kaluretic activity. So, in present study we prepared 70 % methanolic extract of *L. pyrotechnica* and evaluated its phytochemical constituents by GC-MS analysis, antioxidant potential and performed both acute and prolonged diuretic activity in male Wistar rats. Additionally we also explored the underlying mechanism of diuresis and performed the carbonic anhydrase inhibition assay.

Phytochemical analysis of Lp.Cr by GC-MS identified 104 compounds from different chemical classes. Among these, 64 compounds having Qual factor greater than 90 are presented in Table 2. Several polyunsaturated fatty acids, monounsaturated fatty acids, straight chain fatty alcohols, acyclic ditrepene alcohol and phenols were identified. These include methyl lineoleate, methyl alpha-linolenate, Erucic acid, octacosanol, phytol and 2,4-di-*tert*-butylphenol. These compounds have already been reported for their beneficial effects on health, possessing antioxidant activity and used as supplements in various nutraceuticals [[53], [54], [55], [56], [57]]. In present study, antioxidant activity of Lp.Cr was assessed by DPPH, ABTS, nitrite scavenging, CUPRAC and FRAP assays which indicated good antioxidant activity correlating to total phenolic and flavonoid contents. Total tannin contents of Lp.Cr were 0.54 ± 0.01 mg TAE/g dry weight of the extract. Tannins exhibit good antibacterial and antioxidant activity that can contribute ameliorating effect in urinary tract disorders [58]. Total saponin contents of Lp.Cr were also quantified which indicated that 7.93 ± 0.85 % saponins were present in 1 g of Lp.Cr. Many plants have been reported having diuretic activity due to the presence of secondary metabolites in plants. Diuretic action cannot be attributed to any specific functional group or class of plant secondary metabolites. For instance terpenes, phenolics, saponins and alkaloids have reported to have diuretic activity indicating that wide variety of phytochemicals can produce diuretic action [59,60].

Since long many medicinal plants has been used traditionally to control hypertension by reducing blood volume through induction of diuresis [61]. Plants can induce diuresis by several mechanisms such as effect on aquaporins, osmosis, solute transport, prostaglandins, carbonic anhydrase inhibition, NO-pathway and RAAS-pathway [62]. In this study, Lp.Cr increased urinary volume at the dose of 30, 100 and 300 mg/kg. Maximum urinary volume was observed at the dose of 300 mg/kg which was comparable to furosemide 10 mg/kg. Beside urniary volume, excretion of Na^+^, K^+^ and Cl^−^ was also increased of same doses indicating good saluretic effect. This diuretic effect was observed in both acute 24-h and prolonged 7-days study. To explore the involvement of prostaglandin pathway, animals were pre-treated with indomethacin 5 mg/kg and the diuretic effect of 100 mg/kg was observed. Pre-treatment with indomethacin significantly reduced the urinary volume which was even less than the urine volume observed in control animals. Prostaglandins play an important role in kidney homeostasis by maintaining the kidney blood flow and GFR. Both COX-I and COX-II are expressed in kidney, non-selective inhibition of COX enzymes by indomethacin can result in salt and fluid retention [62]. Involvement of muscarinic receptors was assessed by pretreatment with atropine (1.5 mg/kg), which significantly reduced urine volume in a similar fashion as observed with COX inhibition. Previously, it has been reported that *L. pyrotechnica* inhibited acetylcholinesterase and buterylcholinesterase enzymes [47], thus it can be suggested that cholinergic transmission may play a role in diuretic effect of Lp.Cr. In our experiments, we did not see any decrease in Na^+^ and K^+^ excretion which appears contradictory. This might be due to half-life of indomethacin and atropine which are 4.5 and 5 h respectively, while we checked Na^+^ and K^+^ concentration in 24-h urine sample which might be a limitation of the study. Involvement of NO-pathway was assessed by pre-treatment with l-NAME (60 mg/kg) a nonselective nitric oxide synthase inhibitor. Minor decrease in urine volume was observed at 7-h but it did not completely inhibited the diuretic effect of Lp.Cr (100 mg/kg), indicating that NO-pathway may complement the diuretic activity but don't play a major role in diuretic effect of Lp.Cr. We did not observe any significant difference in urea and creatinine excretion however Lp.Cr treatment increased the urinary pH. It has been reported that several natural products can inhibit carbonic anhydrase enzyme and induce alkaline diuresis [63]. We assesed carbonic anhydrase inhibitory activity of Lp.Cr which showed a good inhibition with IC_50_ 2.89 μg/ml thus explaining an increase in urinary pH with Lp.Cr treatment.

## 5 Conclusion

*Leptadenia pyrotechnica* Forssk. Decne. is a member of family Apocynaceae, widely found in sandy and dry habitat in several regions. Its 70 % methanolic extract contained good phenolic and flavonoid contents with good antioxidant activity. GC-MS analysis identified 104 compounds from several phytochemical classes. Plant also exhibited good diuretic, saluretic and carbonic anhydrase inhibitory activity, corroborating its traditional use and making it a potential target for future drug development.

## Data availability statement

All data generated or analyzed has been included in this study.

## CRediT authorship contribution statement

**Noreena Masood:** Data curation, Formal analysis, Investigation. **QurratUlAin Jamil:** Conceptualization, Visualization, Writing – original draft. **Muhammad Irfan Aslam:** Data curation, Formal analysis, Investigation. **Muhammad Irfan Masood:** Data curation, Methodology, Writing – review & editing. **Jafir Hussain Shirazi:** Data curation, Funding acquisition, Methodology, Visualization. **Qazi Adnan Jamil:** Data curation, Funding acquisition, Methodology. **Muhammad Saeed Jan:** Data curation, Formal analysis, Investigation. **Bader Alsuwayt:** Data curation, Funding acquisition, Methodology, Visualization. **Ashfaq Ahmad:** Data curation, Funding acquisition, Visualization, Writing – review & editing. **Sulaiman Mohammed Abdullah Alnasser:** Data curation, Funding acquisition, Visualization, Writing – review & editing. **Mohammed Aufy:** Conceptualization, Funding acquisition, Visualization, Writing – review & editing. **Shahid Muhammad Iqbal:** Conceptualization, Methodology, Supervision, Writing – original draft, Writing – review & editing.

## Declaration of competing interest

The authors declare that they have no known competing financial interests or personal relationships that could have appeared to influence the work reported in this paper.
