# Supplementary material for: Antioxidant, carbonic anhydrase inhibition and diuretic activity of Leptadenia pyrotechnica Forssk. Decne
Source: Heliyon. 2023 Nov 17;9(12):e22485. doi: 10.1016/j.heliyon.2023.e22485 (PMC10709400; doi:10.1016/j.heliyon.2023.e22485)
Supplement: Multimedia component 1 [file mmc1.docx]

Supplementary material

Table 1. Total phytochemicals identified by the GC-MS analysis of 70% methanolic extract of Lp.Cr.

| Peak no | RT | Area% | Compound name | M.F. | M.W. g/mol | Qual |
| --- | --- | --- | --- | --- | --- | --- |
| 1 | 7.43 | 0.04 | 2-methyl-dodecane | C_13_H_28_ | 184.3 | 93 |
| 2 | 7.53 | 0.09 | 2,6,11-trimethyl-dodecane | C_15_H_32_ | 212.4 | 59 |
| 3 | 7.87 | 0.38 | Tridecane | C_13_H_28_ | 184.3 | 97 |
| 4 | 8.08 | 0.28 | Octacosane | C_28_H_58_ | 394.8 | 72 |
| 5 | 8.21 | 0.14 | 2,3-dimethyl-2-butyl-borane | C_12_H_30_B_2_ | 196.2 | 78 |
| 6 | 8.28 | 0.11 | 1,54-dibromo-tetrapentacontane | C_54_H_108_Br_2_ | 917.2 | 74 |
| 7 | 8.35 | 0.12 | 2-hexyl-1-decanol | C_16_H_34_O | 242.4 | 53 |
| 8 | 8.47 | 0.47 | Tetradecyl dichloroacetic acid | C_16_H_30_Cl_2_O_2_ | 325.3 | 46 |
| 9 | 8.57 | 0.27 | 10-methylnonadecane | C_20_H_42_ | 282.5 | 72 |
| 10 | 8.64 | 0.34 | Octacosane | C_28_H_58_ | 394.8 | 80 |
| 11 | 8.72 | 0.37 | Tritetracontane | C_43_H_88_ | 605.2 | 58 |
| 12 | 8.80 | 0.58 | 2,6,10-trimethyldodecane | C_15_H_32_ | 212.4 | 91 |
| 13 | 9.01 | 0.79 | Ethylcyclododecane | C_14_H_28_ | 196.3 | 92 |
| 14 | 9.09 | 1.85 | Tetradecane | C_14_H_30_ | 198.3 | 98 |
| 15 | 9.20 | 0.52 | Tetradecane-6-sulfonic-acid-butyl ester | C_18_H_38_O_3_S | 334.6 | 58 |
| 16 | 9.27 | 0.27 | Tetratetracontane | C_44_H_90_ | 619.2 | 64 |
| 17 | 9.32 | 0.42 | 4-methoxy-6-fluoro-benzyl alcohol | C_8_H_9_FO_2_ | 156.1 | 90 |
| 18 | 9.49 | 1.57 | 2,3-dimethylnaphthalene | C_12_H_12_ | 156.2 | 96 |
| 19 | 9.71 | 1.12 | 5-methyl-tetradecane | C_15_H_32_ | 212.4 | 83 |
| 20 | 9.83 | 1.39 | Tridecane | C_13_H_28_ | 184.3 | 90 |
| 21 | 9.93 | 0.65 | 3-methyl-tetradecane | C_15_H_32_ | 212.4 | 89 |
| 22 | 10.02 | 0.79 | 1,54-dibromo-tetrapentacontane | C_54_H_108_Br_2_ | 917.2 | 25 |
| 23 | 10.17 | 0.33 | Z-8-hexadecene | C_16_H_32_ | 224.4 | 70 |
| 24 | 10.29 | 1.85 | Pentadecane | C_15_H_32_ | 212.4 | 97 |
| 25 | 10.37 | 0.48 | Nonahexacontanoic acid | C_69_H_138_O_2_ | 999.8 | 68 |
| 26 | 10.46 | 1.27 | 2,4-ditert-butylphenol | C_14_H_22_O | 206.3 | 95 |
| 27 | 10.71 | 0.62 | Ethylpentamethyl-benzene | C_13_H_20_ | 176.3 | 53 |
| 28 | 10.81 | 0.47 | 1,6,7-trimethylnaphthalene | C_13_H_14_ | 170.2 | 96 |
| 29 | 10.91 | 0.58 | Decyl-cyclopentane | C_15_H_30_ | 210.4 | 83 |
| 30 | 11.00 | 0.55 | 1,6,7-trimethylnaphthalene | C_13_H_14_ | 170.2 | 96 |
| 31 | 11.09 | 0.36 | 3-methylpentadecane | C_16_H_34_ | 226.4 | 96 |
| 32 | 10.91 | 0.58 | Decyl-cyclopentane | C_15_H_30_ | 210.4 | 83 |
| 33 | 11.34 | 0.46 | Pentadecyl-2-chloroacetate | C_17_H_33_ClO_2_ | 304.9 | 94 |
| 34 | 11.43 | 1.17 | Hexadecane | C_16_H_34_ | 226.4 | 99 |
| 35 | 11.59 | 0.24 | 3,8-dimethyl-decane | C_12_H_26_ | 170.3 | 40 |
| 36 | 11.71 | 0.41 | 3,8-dimethoxy-1,2-naphthoquinone | C_12_H_10_O_4_ | 218.2 | 44 |
| 37 | 11.98 | 0.67 | 2-Bromododecane | C_12_H_25_Br | 249.2 | 95 |
| 38 | 12.08 | 0.45 | 2-Benzylideneheptanal | C_14_H_18_O | 202.2 | 97 |
| 39 | 12.22 | 0.22 | 3-methylhexadecane | C_17_H_36_ | 240.5 | 94 |
| 40 | 12.40 | 0.30 | 1,2-dihydro-1-acenaphthylenol | C_12_H_10_O | 170.2 | 59 |
| 41 | 12.55 | 0.66 | Heptadecane | C_17_H_36_ | 240.5 | 98 |
| 42 | 12.61 | 0.47 | 2,6,10,14-tetramethylpentadecane | C_19_H_40_ | 268.5 | 93 |
| 43 | 12.72 | 0.16 | 2-cyclohexyl-1H-indole | C_14_H_17_N | 199.2 | 27 |
| 44 | 12.82 | 0.25 | 2-Methyl-Z-4-tetradecene | C_15_H_30_ | 210.4 | 90 |
| 45 | 12.91 | 0.16 | 2-Methylthio-5-nitro anisole | C_8_H_9_NO_3_S | 199.2 | 38 |
| 46 | 13.01 | 0.28 | 3-methyl-heptadecane | C_18_H_38_ | 254.5 | 70 |
| 47 | 13.10 | 0.19 | 1-chloro-hexadecane | C_16_H_33_Cl | 260.8 | 60 |
| 48 | 13.16 | 0.12 | 4-methyl-hexadecane | C_17_H_36_ | 240.5 | 83 |
| 49 | 13.22 | 0.2 | 2-methyl-heptadecane | C_18_H_38_ | 254.5 | 96 |
| 50 | 13.31 | 0.14 | 3-methyl-heptadecane | C_18_H_38_ | 254.5 | 94 |
| 51 | 13.36 | 0.10 | Azuleno(2,1-b)thiophene | C_12_H_8_S | 184.2 | 46 |
| 52 | 13.56 | 0.56 | 1-octadecene | C_18_H_36_ | 252.5 | 95 |
| 53 | 13.64 | 0.77 | Octadecane | C_18_H_38_ | 254.5 | 98 |
| 54 | 13.75 | 0.48 | 3-methyl-heptadecane | C_18_H_38_ | 254.5 | 90 |
| 55 | 13.93 | 0.14 | Isopropyl myristate | C_17_H_34_O_2_ | 270.5 | 47 |
| 56 | 14.03 | 0.13 | 13-tetradecen-1-ol acetate | C_16_H_30_O_2_ | 254.4 | 41 |
| 57 | 14.11 | 0.24 | 2-methyl-dodecane | C_13_H_28_ | 184.3 | 78 |
| 58 | 14.18 | 0.19 | 2-decyl-3-(5-methylhex-5-enyl)oxirane | C_19_H_36_O | 280.5 | 49 |
| 59 | 14.29 | 0.10 | Nonahexacontanoic acid | C_69_H_138_O_2_ | 999.8 | 76 |
| 60 | 14.36 | 0.17 | Cembrane | C_20_H_40_ | 280.5 | 94 |
| 61 | 14.45 | 0.17 | 3-methyloctadecane | C_19_H_40_ | 268.5 | 91 |
| 62 | 14.52 | 0.14 | 3-methyl-dibenzothiophene | C_13_H_10_S | 198.2 | 55 |
| 63 | 14.59 | 0.18 | 1-eicosene | C_20_H_40_ | 280.5 | 90 |
| 64 | 14.71 | 0.16 | 2-methyl-heptadecane | C_18_H_38_ | 254.5 | 70 |
| 65 | 14.80 | 0.57 | Nonadecane | C_19_H_40_ | 268.5 | 97 |
| 66 | 14.89 | 0.19 | 13-tetradecen-1-ol acetate | C_16_H_30_O_2_ | 254.4 | 70 |
| 67 | 15.13 | 0.78 | Methyl-hexadecanoate | C_17_H_34_O_2_ | 270.5 | 98 |
| 68 | 15.31 | 0.26 | 1-methyl-anthracene | C_15_H_12_ | 192.2 | 86 |
| 69 | 15.42 | 0.08 | 2,6-dimethylnon-1-en-3-yn-5-yl pentanoate | C_16_H_26_O_2_ | 250.3 | 51 |
| 70 | 15.50 | 0.09 | Heptadecane | C_17_H_36_ | 240.5 | 89 |
| 71 | 15.57 | 0.08 | α-d-glucofuranosyl benzenesulfonate | C_12_H_16_O_8_S | 320.3 | 95 |
| 72 | 15.68 | 0.49 | Dibutyl phthalate | C_16_H_22_O_4_ | 278.3 | 50 |
| 73 | 15.82 | 0.13 | 1-nonadecene | C_19_H_38_ | 266.5 | 93 |
| 74 | 15.98 | 0.89 | 5-eicosene | C_20_H_40_ | 280.5 | 98 |
| 75 | 16.06 | 0.71 | Eicosane | C_20_H_42_ | 282.5 | 99 |
| 76 | 16.17 | 0.14 | Nonadecane | C_19_H_40_ | 268.5 | 86 |
| 77 | 16.26 | 0.12 | 2,4'-dihydroxy-stilbene | C_14_H_12_O_2_ | 212.2 | 55 |
| 78 | 16.38 | 0.26 | 3-deoxyestradiol | C_18_H_24_O | 256.3 | 55 |
| 79 | 16.55 | 0.36 | Pentadecane | C_15_H_32_ | 212.4 | 92 |
| 80 | 16.70 | 0.08 | 1-docosene | C_22_H_44_ | 308.6 | 80 |
| 81 | 16.77 | 0.16 | 1,7-dimethyl-phenanthrene | C_16_H_14_ | 206.2 | 64 |
| 82 | 16.86 | 0.20 | Octadecane | C_18_H_38_ | 254.5 | 93 |
| 83 | 16.98 | 0.13 | Cycloeicosane | C_20_H_40_ | 280.5 | 93 |
| 84 | 17.04 | 0.17 | 2,6,10,14-tetramethyl-hexadecane | C_20_H_42_ | 282.5 | 91 |
| 85 | 17.19 | 0.46 | cis-1-Chloro-9-octadecene | C_18_H_35_Cl | 286.9 | 98 |
| 86 | 17.36 | 1.18 | Methyl lineoleate | C_19_H_34_O_2_ | 294.5 | 99 |
| 87 | 17.48 | 2.03 | 9,12,15-octadecatrienoic acid | C_18_H_30_O_2_ | 278.4 | 96 |
| 88 | 17.63 | 1.28 | Phytol | C_20_H_40_O | 296.5 | 94 |
| 89 | 17.76 | 0.19 | Methyl octadecanoate | C_19_H_38_O_2_ | 298.5 | 95 |
| 90 | 17.90 | 0.41 | 1-chloro-Octadecane | C_18_H_37_Cl | 288.9 | 91 |
| 91 | 18.39 | 6.44 | Methyl alpha-linolenate | C_19_H_32_O_2_ | 292.5 | 91 |
| 92 | 18.43 | 2.73 | Linolenyl alcohol | C_18_H_32_O | 264.4 | 93 |
| 93 | 18.71 | 1.33 | 1-docosene | C_22_H_44_ | 308.6 | 94 |
| 94 | 18.79 | 0.98 | Eicosane | C_20_H_42_ | 282.5 | 97 |
| 95 | 19.22 | 0.32 | Linolenyl alcohol | C_18_H_32_O | 264.4 | 87 |
| 96 | 19.29 | 0.48 | Nonadecane | C_19_H_40_ | 268.5 | 92 |
| 97 | 19.41 | 0.28 | 2-methyl-octadecane | C_19_H_40_ | 268.5 | 91 |
| 98 | 19.58 | 0.56 | Tricosane | C_23_H_48_ | 324.6 | 86 |
| 99 | 19.65 | 0.39 | Docosane | C_22_H_46_ | 310.6 | 95 |
| 100 | 19.79 | 0.42 | Heneicosane | C_21_H_44_ | 296.6 | 96 |
| 101 | 19.94 | 0.53 | 1,3,5-tris(cyclohexyl)pent-1-ene | C_23_H_40_ | 316.6 | 78 |
| 102 | 20.18 | 0.96 | Heptadecane | C_17_H_36_ | 240.5 | 96 |
| 103 | 20.31 | 0.33 | 1,54-dibromo-tetrapentacontane | C_54_H_108_Br_2_ | 917.2 | 81 |
| 104 | 20.69 | 0.37 | Heneicosane | C_21_H_44_ | 296.6 | 92 |
| 105 | 20.78 | 0.21 | Docosane | C_22_H_46_ | 310.6 | 90 |
| 106 | 20.83 | 0.20 | Docosane | C_22_H_46_ | 310.6 | 91 |
| 107 | 21.01 | 0.23 | Heneicosane | C_21_H_44_ | 296.6 | 86 |
| 108 | 21.08 | 0.23 | Tricosane | C_23_H_48_ | 324.6 | 94 |
| 109 | 21.22 | 0.42 | Tetracosane | C_24_H_50_ | 338.7 | 95 |
| 110 | 21.30 | 0.27 | 1-Hexacosene | C_26_H_52_ | 364.7 | 95 |
| 111 | 21.41 | 0.19 | 7-bromomethyl-pentadec-7-ene | C_16_H_31_Br | 303.3 | 83 |
| 112 | 21.56 | 1.19 | Cyclotetracosane | C_24_H_48_ | 336.6 | 99 |
| 113 | 21.63 | 0.6 | Tetracosane | C_24_H_50_ | 338.7 | 98 |
| 114 | 21.74 | 0.38 | Tricosane | C_23_H_48_ | 324.6 | 93 |
| 115 | 21.89 | 0.16 | 1-eicosene | C_20_H_40_ | 280.5 | 95 |
| 116 | 21.96 | 0.30 | 1-Hexacosene | C_26_H_52_ | 364.7 | 99 |
| 117 | 22.12 | 0.41 | Tricosane | C_23_H_48_ | 324.6 | 91 |
| 118 | 22.26 | 0.16 | Docosane | C_22_H_46_ | 310.6 | 91 |
| 119 | 22.45 | 0.38 | Tricosane | C_23_H_48_ | 324.6 | 93 |
| 120 | 22.51 | 0.20 | Docosane | C_22_H_46_ | 310.6 | 93 |
| 121 | 22.66 | 0.32 | Tricosane | C_23_H_48_ | 324.6 | 95 |
| 122 | 22.78 | 0.31 | 1-hexacosene | C_26_H_52_ | 364.7 | 93 |
| 123 | 22.89 | 0.28 | 2,6,10,14,18-eicosapentaene | C_25_H_42_ | 342.6 | 90 |
| 124 | 23.05 | 0.49 | Pentacosane | C_25_H_52_ | 352.7 | 98 |
| 125 | 23.15 | 0.57 | Erucic acid | C_22_H_42_O_2_ | 338.6 | 91 |
| 126 | 23.55 | 0.54 | Docosane | C_22_H_46_ | 310.6 | 95 |
| 127 | 23.93 | 6.74 | 2-ethylhexyl hydrogen phthalate | C_16_H_22_O_4_ | 278.3 | 91 |
| 128 | 24.09 | 0.52 | Tetracosane | C_24_H_50_ | 338.7 | 93 |
| 129 | 24.23 | 0.33 | 1-Hexacosene | C_26_H_52_ | 364.7 | 96 |
| 130 | 24.43 | 1.50 | 1-nonadecene | C_19_H_38_ | 266.5 | 98 |
| 131 | 24.58 | 0.35 | Hexacosane | C_26_H_54_ | 366.7 | 94 |
| 132 | 24.94 | 0.97 | Hexacosane | C_26_H_54_ | 366.7 | 94 |
| 133 | 25.11 | 0.14 | Pentacosane | C_25_H_52_ | 352.7 | 98 |
| 134 | 25.20 | 0.16 | 1-Hexacosene | C_26_H_52_ | 364.7 | 97 |
| 135 | 25.30 | 0.63 | 1-Hexacosene | C_26_H_52_ | 364.7 | 95 |
| 136 | 25.51 | 0.33 | Pentacosane | C_25_H_52_ | 352.7 | 93 |
| 137 | 25.66 | 0.41 | 1-Hexacosene | C_26_H_52_ | 364.7 | 92 |
| 138 | 26.09 | 5.13 | Cis-Permethrin | C_21_H_20_Cl_2_O_3_ | 391.3 | 99 |
| 139 | 26.37 | 5.77 | trans-Permethrin | C_21_H_20_Cl_2_O_3_ | 391.3 | 99 |
| 140 | 26.5 | 0.23 | cyclohexylbis[5-methyl-2-(1-methylethyl)cyclohexyl]- Phosphine | C_26_H_49_P | 392.6 | 91 |
| 141 | 26.58 | 0.39 | 1-Hexacosene | C_26_H_52_ | 364.7 | 59 |
| 142 | 26.76 | 0.51 | Cyclotriacontane | C_30_H_60_ | 420.8 | 96 |
| 143 | 26.88 | 0.62 | Hexacosane | C_26_H_54_ | 366.7 | 93 |
| 144 | 27.21 | 1.98 | 1-nonadecene | C_19_H_38_ | 266.5 | 98 |
| 145 | 27.60 | 0.28 | 1-Hexacosene | C_26_H_52_ | 364.7 | 95 |
| 146 | 27.70 | 0.71 | 1,54-dibromo-tetrapentacontane | C_54_H_108_Br_2_ | 917.2 | 90 |
| 147 | 28.04 | 0.40 | 1-(4-bromobutyl)-2-piperidinone | C_9_H_16_BrNO | 234.1 | 91 |
| 148 | 28.10 | 0.36 | Cyclotriacontane | C_30_H_60_ | 420.8 | 95 |
| 149 | 28.25 | 0.34 | Pentacosane | C_25_H_52_ | 352.7 | 56 |
| 150 | 28.44 | 0.27 | 2-dodecen-1-ylsuccinic anhydride | C_16_H_26_O_3_ | 266.3 | 93 |
| 151 | 28.60 | 0.68 | Heptacosane | C_27_H_56_ | 380.7 | 93 |
| 152 | 29.01 | 0.99 | Octacosane | C_28_H_58_ | 394.8 | 95 |
| 153 | 29.46 | 0.41 | Hexacosane | C_26_H_54_ | 366.7 | 90 |
| 154 | 29.91 | 0.60 | Octacosanol | C_28_H_58_O | 410.8 | 95 |
| 155 | 30.41 | 0.20 | Triacontane | C_30_H_62_ | 422.8 | 95 |
| 156 | 30.89 | 0.34 | 28-nor-17.beta.(H)-hopane | C_29_H_50_ | 398.7 | 83 |
| 157 | 30.99 | 0.13 | 1-Hexacosene | C_26_H_52_ | 364.7 | 94 |
| 158 | 31.53 | 0.23 | Octacosane | C_28_H_58_ | 394.8 | 93 |
| 159 | 32.09 | 0.23 | Eicosane | C_20_H_42_ | 282.5 | 96 |
| 160 | 32.33 | 0.29 | 28-nor-17.beta.(H)-hopane | C_29_H_50_ | 398.7 | 91 |
| 161 | 33.38 | 0.33 | 1-Hexacosene | C_26_H_52_ | 364.7 | 99 |
| 162 | 34.07 | 0.16 | Eicosane | C_20_H_42_ | 282.5 | 95 |
| 163 | 34.32 | 0.26 | Pyridine-3-carboxamide | C_13_H_10_F_3_N_3_O | 281.2 | 91 |
| 164 | 34.59 | 0.10 | Friedelan-3-one | C_30_H_50_O | 426.7 | 53 |
| 165 | 35.73 | 0.10 | Cyclotriacontane | C_30_H_60_ | 420.8 | 90 |
| 166 | 36.16 | 0.26 | Cyclotriacontane | C_30_H_60_ | 420.8 | 50 |
| 167 | 37.57 | 0.52 | Isomultiflorenon | C_30_H_48_O | 424.7 | 89 |
